# Longitudinal self‐administered smartphone assessments capture cognitive decline and disease progression in presymptomatic and early symptomatic FTLD

**DOI:** 10.1002/alz.090579

**Published:** 2025-01-09

**Authors:** Mark E. Sanderson‐Cimino, Emily W. Paolillo, Amy B. Wise, Sreya Dhanam, Jack C. Taylor, Hilary W. Heuer, Jason J. Hassenstab, Mai Anh Bui, J. Clayton Young, Kaitlin B. Casaletto, Brandon R. Palacios, Rowan Saloner, Leah K. Forsberg, John Kornak, Walter K. Kremers, Sandra Weintraub, Katya Rascovsky, Katherine P. Rankin, Bonnie Wong, Julie A. Fields, Joel H. Kramer, Brad F. Boeve, Howard J. Rosen, Adam L. Boxer, Adam M. Staffaroni

**Affiliations:** ^1^ Memory and Aging Center, UCSF Weill Institute for Neurosciences, University of California, San Francisco, San Francisco, CA USA; ^2^ Memory and Aging Center, UCSF Weill Institute for Neurosciences, San Francisco, CA USA; ^3^ Knight Alzheimer Disease Research Center, St. Louis, MO USA; ^4^ Mayo Clinic, Rochester, MN USA; ^5^ Northwestern University Feinberg School of Medicine, Chicago, IL USA; ^6^ Penn FTD Center, Perelman School of Medicine, University of Pennsylvania, Philadelphia, PA USA; ^7^ Massachusetts General Hospital, Harvard Medical School, Boston, MA USA

## Abstract

**Background:**

Many treatments targeting frontotemporal lobar degeneration (FTLD) are in the developmental pipeline, but the rarity of the disease, coupled with the behavioral and motor features of FTLD, make it challenging to identify sufficient trial participants who can attend frequent in‐person visits. Decentralized clinical trial designs with remote evaluations are attractive alternatives but require validated tools for symptom tracking. Our previous cross‐sectional analyses showed that cognitive tasks deployed via the ALLFTD Mobile App are reliable and sensitive to early stages of disease. The current study seeks to evaluate whether unsupervised smartphone cognitive tests are sensitive to longitudinal changes that occur with increasing FTLD disease stage.

**Method:**

The study included 194 participants (mean baseline age 54.30, SD = 15.66; mean education = 16.52, SD = 2.31; 55.2% women) who were asymptomatic (Clinical Dementia Rating plus FTLD (CDR®+NACC‐FTLD) = 0 [n = 109; 43 carriers of FTLD‐associated genetic variants]), prodromal (CDR®+NACC‐FTLD = 0.5 [n = 39]), or symptomatic (CDR®+NACC‐FTLD>0.5 [n = 46]) at baseline. Participants completed ALLFTD Mobile App smartphone versions of Stroop, flanker, and 2‐back tasks, as well as an adaptive associative memory task, every six months for up to two years. Linear‐mixed effects models investigated the effect of disease severity on cognitive trajectories by testing the interaction of baseline CDR®+NACC‐FTLD box score and time on smartphone test scores. Analyses were repeated in the subsample of asymptomatic/prodromal CDR®+NACC‐FTLD<1) familial FTLD mutation carriers (*C9orf72*, n = 27; *MAPT*, n = 16). All models controlled for age, education, and sex.

**Result:**

Greater baseline disease severity was associated with lower cognitive scores across all tasks (β range: ‐0.28 to ‐0.13; *p’*s < 0.001), and faster rates of decline on the memory and 2‐back tasks (β range: ‐0.15 to ‐0.07; *p*’s<0.04). When the sample was restricted to asymptomatic and prodromal individuals (CDR®+NACC FTLD<1), greater baseline disease severity was still associated with lower scores across most tasks (β range: ‐0.48 to ‐0.27; *p*’s<0.002; 2‐back: β = ‐0.19, *p* = 0.26). When the sample was restricted to asymptomatic/prodromal mutation carriers, disease severity remained associated with the memory and flanker tasks (β range: ‐0.85 to ‐0.45; *p*’s<0.01).

**Conclusion:**

This study provides initial evidence that longitudinal smartphone‐based cognitive measurements capture the accelerated cognitive worsening that accompanies increasing disease progression in FTLD.

